# Supplementary material for: Estimating the effectiveness of non-pharmaceutical interventions against COVID-19 transmission in the Netherlands
Source: PLoS Comput Biol. 2025 Sep 17;21(9):e1013502. doi: 10.1371/journal.pcbi.1013502 (PMC12459772; doi:10.1371/journal.pcbi.1013502)
Supplement: Attachment — Submitted filename: Response_to_reviewers.pdf [file pcbi.1013502.s003.pdf]

Response to review on

PCOMPBIOL-D-25-00738
Estimating the effectiveness of non-pharmaceutical interventions against COVID-19 transmission in the Netherlands

We thank the reviewers for critically assessing our manuscript. Below we have responded to the comments in italics. Line numbers refer to the revised manuscript with tracked changes.

To note: we have updated our results according to the updated results of the estimation of seasonal effects, which is now accepted for publicaton in J R Soc Interface (doi: 10.1098/rsif.2025.0317). The estimated reduction of the reproduction number in summer compared to winter is 41% instead of 33% (translating to a sinusoidal amplitude of 26% instead of 20%). As a consequence, text (lines 131, 222, 240, 271, 283, 428, 430), figures (1, 5 and 6) and table 1 needed to be updated, but this did not change the overall results or conclusions.

Reviewers' comments:

Reviewer's Responses to Questions

Reviewer #1: Summary

This manuscript presents an approach to estimate the effectiveness of non-pharmaceutical interventions (NPIs) on COVID-19 transmission in the Netherlands from February 2020 to October 2021. By integrating multiple data sources (including reproduction number estimates, genomic surveillance, vaccination coverage, and seroprevalence surveys), the authors simulate time-varying counterfactual reproduction numbers in the absence of NPIs. They quantify the reduction in transmission attributable to NPIs, accounting for three components: (1) seasonality, (2) variant emergence, and (3) population immunity. Overall, the manuscript is methodologically strong and timely. A few clarifications and minor revisions are suggested below.

Major Comments

The counterfactual reproduction number is constructed under the assumption of linear scaling with the immune fraction and multiplicative effects from variant transmissibility and seasonal variation. Please acknowledge that this approach may not fully capture heterogeneities such as contact structure or setting-specific transmission dynamics.

We agree that the counterfactual reproduction number is a simplification based on population averages and linear scaling. We now make these underlying assumptions more explicit by adding: "By using the population average of the immune fraction phi(t) we disregard any heterogeneities in, for instance, contact structure or setting-specific transmission." (lines 105-106) and "We model the seasonal fluctuations sigma(t) and the relative transmissibility theta(t) of circulating variants as multiplicative effects on the basic reproduction number:" (lines 115-116). The case of heterogeneity in contact structure is discussed as first limitation of our method (lines 330-335).

The manuscript introduces a time-varying basic reproduction number, which diverges from the standard epidemiological definition of R0 as a constant. This may cause confusion, especially given the presence of a separate constant "initial R0". Consider renaming the basic reproduction number to avoid confusion.

We understand that our use of a time-varying basic reproduction number can be confusing. We need to explain better that R0 is not a constant but depends on the population (structure) and environmental circumstances at the time of introduction (Keeling & Rohani, 2011). We have interpreted these environmental circumstances to include the seasonal effect and the variants circulating at a specific time. In our opinion this is still in line with the basic concept of R0 (number of secondary infections per typically infected in a susceptible population), and for this reason we think the term "time-varying basic reproduction number" describes the variable accurately.

After the definition "The basic reproduction number R_0 is defined as the average number of secondary infections caused by one infected person in a fully susceptible population without any interventions." (lines 110-111), we have added: "We allow the basic reproduction number to vary over time depending on the circumstances at time t [@Keeling_2011; @Delamater_2019], specifically on the time of year and on the circulating variants." (lines 111-114)

Changed in introduction: "Firstly, circumstances can change over time because of the emergence of more transmissible variants [@Campbell_2021] or because of seasonal effects [@Xu_2020; @Gavenciak_2022; @Paireau_2023; @Klinkenberg_2025]" (lines 78-80)

Minor Comments

Line 40: Remove "(and references therein)"

Deleted as suggested.

Line 110: "lower than winter"

Changed, thank you for pointing this out.

Line 135: "October 2021."

Changed, thank you for pointing this out.

Figure 1: The 95% confidence intervals for vaccination lines are not available. Either add uncertainty or update the legend to reflect what is shown.

The vaccinated fraction in Fig.2 reflects the actual numbers of administered vaccinations and hence lack a confidence interval. We have clarified the calculation in the figure caption by replacing "The vaccination data per week and age group was linearly interpolated to obtain the vaccinated fraction per day and age group" by "The vaccinated fraction per day and age group was obtained by dividing the reported number of administered vaccinations per week and age group by the population size per age group and linear interpolation." (lines 161-164)

Figure 4: The right panel shows shaded areas for observed breakthrough infections, but the left panel lacks this uncertainty. For consistency, clarify which lines include uncertainty intervals and which do not.

We have expanded the figure caption by replacing "Observed infections in partly vaccinated persons are counted for 0% (lower limit of grey area), 50% (mean) or 100% (upper limit of grey area) as breakthrough infection." by "The observed fraction of breakthrough infections (right panel) depends on whether infections in partly vaccinated persons are counted for 0% (lower limit of grey area), 50% (mean) or 100% (upper limit of grey area) as breakthrough infection. The observed fraction of

reinfections (left panel) lacks this uncertainty." (lines 210-213)

Figures 5 and 6: Display the negative y-axis to reflect effectiveness values below 0%, as mentioned in line 200. Also, include a legend explaining the values represented by the stringency index color band.

We have now included the negative y-axis in Figs 5 and 6. As for the actual values of the stringency index, we don't think that this has an added value for the following reason. The stringency index is a composite measure of different interventions with each a designated value that has no physical interpretation. Different sets of interventions can have a similar stringency index but are not necessarily equally stringent. In our opinion it is more instructive to consider changes in stringency index over time that do indicate a higher stringency (when interventions are added) or lower stringency (when interventions are lifted). This is what is expressed by the color band that shows the changes over time, and which on purpose makes it impossible to compare the dark red color in March/April 2020 with the dark red color in Jan/Feb 2021. As we do understand that some readers may be more familiar with the absolute values of the stringency index, we added the extreme values and an intermediate value in the caption as reference: "As a timeline reference the Oxford Stringency Index [@Hale_2021] is depicted as a colour band from low stringency (darkest blue, index of 0) via intermediate (white, index of 41) to high (darkest red, index of 82)." (lines 268-270 and 286-288)

Line 280: Suggest explicitly stating the months instead of using "winter 2020/2021"

Changed to "January/February 2021" (lines 321 and 323) and changed to "the first months of 2021" (lines 226)

Line 287: "population level"

Changed, thank you for pointing this out.

Lines 365 and 437: Clarify how validation data were used more clearly.

The validation data is only used in Fig. 4 to visually compare the fractions obtained from our reconstruction with the reported fractions. We have extended the explanation in Methods: "For validation, we calculate the fraction of new infections that were previously infected (reinfections) and vaccinated (breakthrough infections) in the full population. We compare these fractions to the external validation data based on the vaccination status and infection type of reported test-positive cases by plotting them over time. We visually compare the rate and the extent to which the modelled and reported fractions increase." (lines 486-490)

Table 1: Update the reference column to use the [number] format consistent with in-text citations. Ensure all cited studies are included each row. Specify whether waning rates per day were taken directly from referenced studies or derived by the authors based on those studies.

We have replaced the abbreviated references with the in-text citation format. We added an additional reference for the peak time of the seasonality effect (1 Feb). For parameters that we assumed, we now indicate "assumed" in the reference column. The waning rates were derived from the referenced studies; the equation is shown in Suppl. S2.3, which is now mentioned in line 475.

Reviewer #2: The manuscript titled "Estimating the effectiveness of NPIs against COVID-19 transmission in the Netherlands" quantifies the benefits of NPIs in real time by showing how NPIs lead to decrease in the effective reproduction number.

The manuscript is well-written, and overall I like the work.

I am tempted to suggest acceptance, but would like the authors to respond to some comments/questions.

Minor comments:

Line 62-63: can you further expand on basic reproduction number? By now, there are already three kind of reproduction numbers— effective, counterfactual, and now basic.

We agree mentioning the basic reproduction number in the introduction without explanation is confusing. We now only use effective and counterfactual reproduction numbers in the introduction, by changing: "Firstly, circumstances can change over time because of the emergence of more transmissible variants [@Campbell_2021] or because of seasonal effects [@Xu_2020; @Gavenciak_2022; @Paireau_2023; @Klinkenberg_2025]" (lines 78-80)." The basic reproduction number is introduced and defined later in the text (lines 110-111).

Furthermore, we try to be more consistent in the use of effective and observed reproduction number, by using "effective reproduction number" by default or "observed effective reproduction number" when we'd like to emphasize that this number can be measured (as opposed to the counterfactual reproduction number). Changed in lines: 17, 57, 68, 217, 255, 421, 422, 425, 495

Line 109-110: I think you meant "…. In summer 33% lower than in winter".

Changed, thank you for pointing this out.

Fig. 2: Please briefly describe in the main text how blue curves have been generated? Are these some sort of curve fittings to 3 data points shown in the plots?

The 3 data points are indeed measurements from the serological survey (the fourth data point is outside the plotted range). In between survey rounds we use the cumulative number of test-positive cases for imputation (i.e., a constant incidence of test-positive cases would yield a linear increase of the fraction in between survey rounds), which is explained in detail in Suppl. S2.2. We have changed the explanation in the caption of Fig.2: "The fraction is measured in survey rounds every few months; the fraction in between survey rounds is imputed using the daily cumulative number of test-positive cases per age group in between survey rounds (see Suppl. S2.2)." (lines 158-161). We prefer to have the explanation on the data (also for the vaccination data) in the caption of the data figure, as not to interrupt the flow of the main text.

In Fig.3: Has any equation being used to plot the curves? If so, please mention it in the main text. Or is it purely data analysis and interpolation?

The reconstruction of the immune fraction in each age group in Fig. 3 is the result of combining the data with immunity waning rates; the equations for the reconstruction are given in Suppl. S2. This is mentioned in "With these waning rates, the immune fraction is reconstructed from the infection and vaccination incidence on each day in each age group (see Methods and Suppl. S2)." (lines 170-172) and we added to the caption of Fig. 3 "The equations for the reconstruction of immune fractions are

given in Suppl. S2." (lines 192-193)

Line 142-144: Please briefly add to the text how you have accounted for immunity waning. For a PloS CB reader, I would tend to expect a bit more detail description in the main text.

We have changed the sentence to: "We reconstructed the immune fraction in each age group and in the full population over time using the data in Figure 2 and assuming immunity wanes exponentially over time, where natural immunity wanes faster than vaccine-induced immunity [@Stein_2023; @Wu_2023]. With these waning rates, the immune fraction is reconstructed from the infection and vaccination incidence on each day in each age group (see Methods and Suppl. S2)." (lines 167-172)

Line 145: may be consider changing naïve to naive (for consistency)

Changed (twice)

Line 152: Please cite some references to support this. Or is it the conclusion from Fig. 3 that vaccine induced immunity wanes faster than natural immunity?

This is the effect of the assumed waning rates, which is now explicitly mentioned: "assuming immunity wanes exponentially over time, where natural immunity wanes faster than vaccine-induced immunity [@Stein_2023; @Wu_2023]." (lines 168-170). To emphasize this we added "(consistent with the assumed waning rates)" (lines 181-182).

Line 163: What are these "calculations"? Are these mathematical calculation? Please clarify on this.

We agree this is a bit vague. We changed "To validate these calculations" to: "To validate the reconstruction of the immune fraction" (line 194).

Line 164: What are breakthrough infections and how are they different from reinfections?

Breakthrough infections are infections in vaccinated individuals, but we agree this may not be a familiar term to all readers. We gave a short explanation in brackets: "reinfections (in previously infected persons) and breakthrough infections (in vaccinated persons)" (lines 195-196).

Line 169: The sentence suggests that there is some underlying model for immunity waning. Can you bring it to the main text and discuss here?

We assume exponential waning for both infection- and vaccine-induced immunity. The equations are given in Suppl. S2.3, but we have added: "assuming immunity wanes exponentially over time, where natural immunity wanes faster than vaccine-induced immunity [@Stein_2023; @Wu_2023]." (lines 168-170) and refer to the supplement in line 475.

Line 193-194: Is it a new result? If not, it will be helpful to cite some references. Same for the sentence 197-198.

In these paragraphs we try to relate our results (estimated effectiveness of NPIs) to the input data (emergence of Alpha, Fig. 1, start of vaccination, Fig. 2) and to the NPIs that were actually in place, i.e., the long lockdown in the first months of 2021 and the reopening of night clubs in July 2021. We have added references to the timeline of NPIs to clarify this (lines 227 and 233).

Line 199: What does negative effectiveness mean? How can NPIs have negative effect?

A negative effectiveness suggests that the NPIs have an adverse effect: instead of decreasing they increase the reproduction number. This is what is thought to have happened in July 2021, when the opening of night clubs sparked many more contacts than 'normal' (without any interventions). We have expanded "the effectiveness dropped below 0%, suggesting an adverse effect of the NPIs" (lines 234-235).

Line 216: Are you particularly referring to Fig. 5B? If so, please update the figure reference accordingly.

Yes, updated

General comments:

Why only restrict to Netherlands? How about comparison with the results of refs. 4-9 that assume constant affect of season etc. through time?

The studies in refs [4-9] aim to quantify the effectiveness of individual interventions, which is based on a fundamentally different approach than ours. The effectiveness of school closure (studies [4-9]) can not be compared to the effectiveness of NPIs in a period when schools were closed (our study) as more interventions were in place in the same period. Our method can be applied to other countries when the required data is available, but we do not (yet) have access to these.

The manuscript is about quantifying (important) the effectiveness of NPIs. We all in some sense agree about the usefulness of NPIs. But what is the cost of NPIs? Can you say anything about that? Example, economic cost, social resistance etc. Any references?

We believe this study on effectiveness offers perspective for future studies into the costs as well as the benefits of NPIs. These costs encompass a wide range of impacts on the economy (unemployment), the health care system (postponing elective surgery), mental health (loneliness), etc, which we made more explicit: "Retrospectively, the results can help to assess the balance between the benefits of the NPIs and the costs, in terms of their impact on, for instance, the economy [@Vardavas_2023] and general wellbeing [@VanDenBoom_2023]." (lines 368-370).

Line 106: For other variants immune escape is also at play. Yet R_0 is defined for those strains. Is it true? Shouldn't immune escape be somehow taken care by the definition of R_0?

R_0 is defined for a fully susceptible population where immune escape does not play a role. We have assumed that the higher growth rates of Alpha and Delta (compared to wildtype and Alpha, respectively) are completely caused by an increase in R_0. If they would also partly escape existing immunity, this would be modelled by a decrease in the immune fraction phi(t) (or more precisely: an increase in susceptibility). For Alpha and Delta it is justified to attribute the higher growth rate to R_0, while for Omicron it is attributed to immune escape. As the decrease of the immune fraction due to immune escape is not straightforward to calculate, we have not included the Omicron period in the analysis.
